# Local motion adaptation enhances the representation of spatial structure at EMD arrays

**DOI:** 10.1371/journal.pcbi.1005919

**Published:** 2017-12-27

**Authors:** Jinglin Li, Jens P. Lindemann, Martin Egelhaaf

**Affiliations:** Department of Neurobiology and Cluster of Excellence Cognitive Interaction Technology (CITEC), Bielefeld University, Bielefeld, Germany; Northeastern University, UNITED STATES

## Abstract

Neuronal representation and extraction of spatial information are essential for behavioral control. For flying insects, a plausible way to gain spatial information is to exploit distance-dependent optic flow that is generated during translational self-motion. Optic flow is computed by arrays of local motion detectors retinotopically arranged in the second neuropile layer of the insect visual system. These motion detectors have adaptive response characteristics, i.e. their responses to motion with a constant or only slowly changing velocity decrease, while their sensitivity to rapid velocity changes is maintained or even increases. We analyzed by a modeling approach how motion adaptation affects signal representation at the output of arrays of motion detectors during simulated flight in artificial and natural 3D environments. We focused on translational flight, because spatial information is only contained in the optic flow induced by translational locomotion. Indeed, flies, bees and other insects segregate their flight into relatively long intersaccadic translational flight sections interspersed with brief and rapid saccadic turns, presumably to maximize periods of translation (80% of the flight). With a novel adaptive model of the insect visual motion pathway we could show that the motion detector responses to background structures of cluttered environments are largely attenuated as a consequence of motion adaptation, while responses to foreground objects stay constant or even increase. This conclusion even holds under the dynamic flight conditions of insects.

## Author summary

Insects, with their limited brain resources and high performance in a wide behavioral repertoire, are exquisite model systems for studying parsimonious signal processing. They extract spatial information by actively shaping their self-motion (e.g. when performing peering movements or during flight segments with fixed gaze) and estimate distance according to the speed of the resulting retinal displacements. The computation of retinal speed is accomplished by arrays of motion detector circuits retinotopically arranged in the second neuropile layer of the visual system. Sharing general adaptive response characteristics with other neurons and neuronal circuits, the responses of motion detectors depend on stimulus history. In the present study, we developed a novel adaptive model of the visual motion pathway of insects and analyzed the consequences of motion adaptation for computing spatial information about the 3D environment. We found that motion adaptation facilitates the segregation of nearby objects from their cluttered background during dynamic locomotion. The functional significance of motion adaptation is likely to generalize to optic flow-based spatial vision in other animals, and the motion adaptation mechanism implemented in our model could also be useful for artificial visual systems.

## Introduction

Spatial vision is a fundamental challenge for animals moving in cluttered environments, and there is no exception for flying insects. Because of their small brains insects have to rely on parsimonious principles to compute spatial information about their environment. Possessing eyes that are close together, binocular spatial vision is no option in the spatial range that is behaviorally relevant for flight control. Alternatively, optic flow, i.e. the displacement of projections of surrounding objects on the retina during an animal’s locomotion, may provide the information needed about the surrounding depth structure. However, optic flow cues only provide depth information during translational self-motion, i.e. self-motion with the gaze direction kept constant over time. During pure rotations the retinal images of surrounding objects are displaced with the same angular velocity irrespective of distance [1]. Insects, such as flies and bees, shape their flight into rapid saccadic turns of head and body and translational segments where the gaze is largely kept constant [1–6]. This behavioral strategy ‘purifies’ the translational flow by separating it from the rotational one and potentially serves the function of simplifying the computation of depth information.

Optic flow is not readily available at the input level of the visual system. Rather, motion detectors are required to compute optic flow information from the spatiotemporal retinal brightness changes induced during locomotion. In the visual systems of insects retinal intensity changes are encoded in membrane-potential changes by arrays of photoreceptors. The photoreceptor responses are band-pass filtered in the first visual neuropile, the lamina. The output of lamina cells is then used to compute local motion in the next neuropile, the medulla (e.g. [7]). Several variants of a particular model of motion detection, the correlation-type elementary motion detector (EMD), have been suggested to account for the functional properties of the insect motion detection circuit [8–10]. As a common feature of all these model variants, motion is detected by correlating the non-delayed signal originating from one retinal input with a temporally delayed signal originating from a neighboring input. This model can successfully explain not only a wide range of electrophysiological data on the large-field motion sensitive lobula plate tangential cells (LPTCs), which spatially pool over arrays of EMDs, but also motion-induced behavior such as optomotor following (review: [9, 11]). With genetic tools, more and more details about the neuronal basis of the motion detector circuits are being unraveled [12–20]. It has been shown in modeling studies that signals represented at the output of EMD arrays correlate well with the contrast-weighted nearness during behaviorally shaped translational self-motion [21, 22].

Like photoreceptors, which adaptively encode light intensities, the neuronal circuits for motion detection are adaptive to motion. Adaptation is a general feature of neurons encoding information about the environment and allows to encode physical parameters that can vary over several decades by neurons with a limited operating range. Moreover, adaptive coding can also reduce redundancies in the sensory input, enhance changes in the signals, and may support energy efficiency of the neural computations [23–25]. Since local motion detectors are difficult to access in electrophysiological experiments, most experimental evidence for adaptation of the motion detection pathway was obtained in LPTCs that are post-synaptic to the local motion detection circuits [26–32]. One major adaptive feature observed in LPTCs is the reduction of the cell responses during constant-velocity motion with retained or even enhanced sensitivity to brief velocity changes [26, 27]. This adaptive feature has been concluded to be generated, to a large extent, pre-synaptically to the LPTCs by a local retinotopic mechanism, although the exact location of this mechanism is still an open question [26].

Cluttered environments cause fluctuations in velocity across the retina under natural flight conditions, especially during translational flight at a constant velocity because of discontinuities in the depth structure of the surroundings. Therefore, we hypothesize that motion adaptation may enhance the representation of spatial information at the level of arrays of motion detectors. Following the same idea, Liang et al. [30] simulated the optic flow experienced by a free-flying fly in a box covered with photographs of a meadow scenery and a black cylinder positioned close to the loop-shaped flight trajectory. By repeatedly presenting this behaviorally generated optic flow to a fly, while recording from an LPTC, the consequences of motion adaptation for representing the cylinder in the neural response could be analyzed. Whereas responses to the walls of the flight arena were reduced by adaptation, the responses to the cylinder remained large [30]. Hence, the wide-field motion sensitive neuron became more sensitive to a nearby object relative to its background as a consequence of adaptation.

In the present study, this hypothesis was systematically tested and validated by model simulations. First, we developed an adaptive model of the visual motion pathway of insects that captures benchmark features of motion adaptation as analyzed in previous electrophysiological studies on LPTCs [26–28]. Our adaptive EMD model is based on an adaptation mechanism similar to the mechanisms previously proposed for light adaptation by photoreceptors [22], here however, operating on the output of EMDs and with much larger time constants. Based on this adaptive model of the visual motion pathway, our intention was to understand how motion adaptation affects the signal representation at the output of arrays of motion detectors and, in particular, the representation of the spatial layout of the environment during translational self-motion in 3D environments. With simulations of an insect model translating in both simple virtual and naturally cluttered 3D environments, we show that by reducing the response to background motion and maintaining large responses to nearby objects, motion adaptation can make nearby objects more salient. The conclusion that motion adaptation facilitates the segregation of nearby objects from their background during translational flight was further validated by taking the natural flight dynamics of insects into account.

## Materials and methods

**Fig 1:**
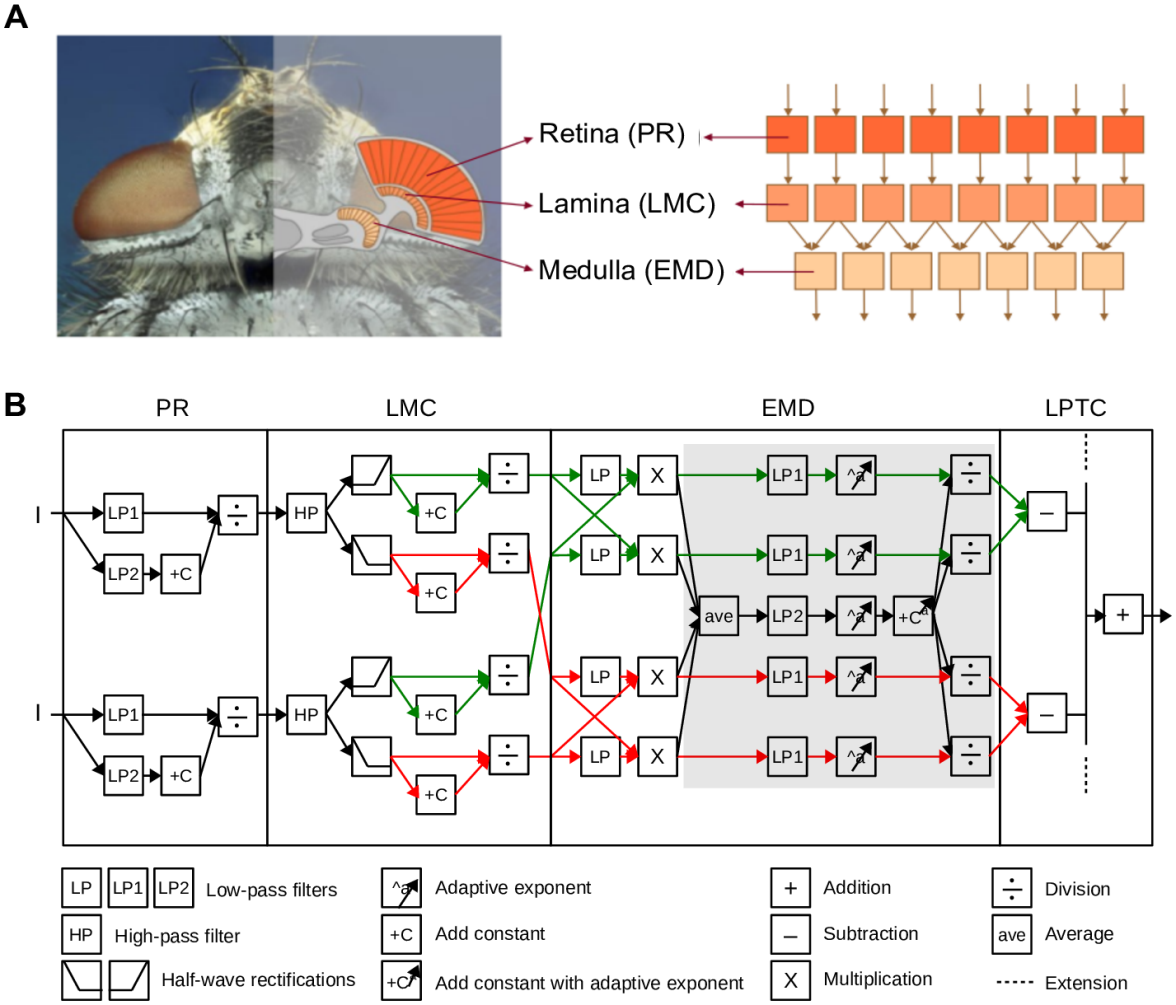
Adaptive model of the visual motion pathway. (A) Schematic illustration of the organization of the insect (fly as an example) visual motion pathway (left), and the retinotopic structure of its model counterpart (right). (B) Computations performed in two neighboring channels of the model. Input light intensity (*I*) is processed at successive stages of (1) the adaptive photoreceptor (*PR*) model, which is realized by dividing a fast signal channel (low-pass filtered with small time constant *PR*.*τ*_*LP*1_) by a slow signal channel (low-pass filtered with large time constant *PR*.*τ*_*LP*2_) in a saturation-like Lipetz transformation; (2) LMC model, which consists of a high-pass filter, a half-wave rectification stage that splits the signal into an ON and an OFF channel, and a saturation-like Lipetz transformation; and (3) adaptive EMD model, which is composed of a basic Hassenstein-Reichhardt detector with a low-pass filter in its cross-channels, the output of which is adapted by dividing a fast branch (low- pass filtered by *EMD*.*τ*_*LP*1_) by a slow one representing motion direction-independent motion energy (average half-detector output low-pass filtered by *EMD*.*τ*_*LP*2_) in a saturation-like Lipetz transformation with adaptive exponent *a* to each component of the transformation (components involved in motion adaptation are overlayed by gray aera); and (4) a simple LPTC model pooling the half-detector output of ON and OFF pathway to preferred and anti-preferred direction over the entire receptive field. Parameters for the PR model: *PR*.*τ*_*LP*1_ = 9*ms*; *PR*.*τ*_*LP*2_ = 250*ms*; *C*_*PR*_ = 10. Parameters for LMC model: *LMC*.*τ*_*HP*_ = 10*ms*; *C*_*LMC*_ = 0.03. Parameters for EMD model: *EMD*.*τ*_*LP*_ = 50*ms*; *EMD*.*τ*_*LP*1_ = 20*ms*; *EMD*.*τ*_*LP*2_ = 4000*ms*; *C*_*EMD*_ = 0.8. *a* is adaptive to the average EMD response before adaptation (i.e. the output of “ave” icon) according to Eq (3). In this equation, *a*_*max*_ = 3; *a*_*min*_ = 0.5; *p*_1_ = 30; *p*_2_ = 150.

**Fig 2:**
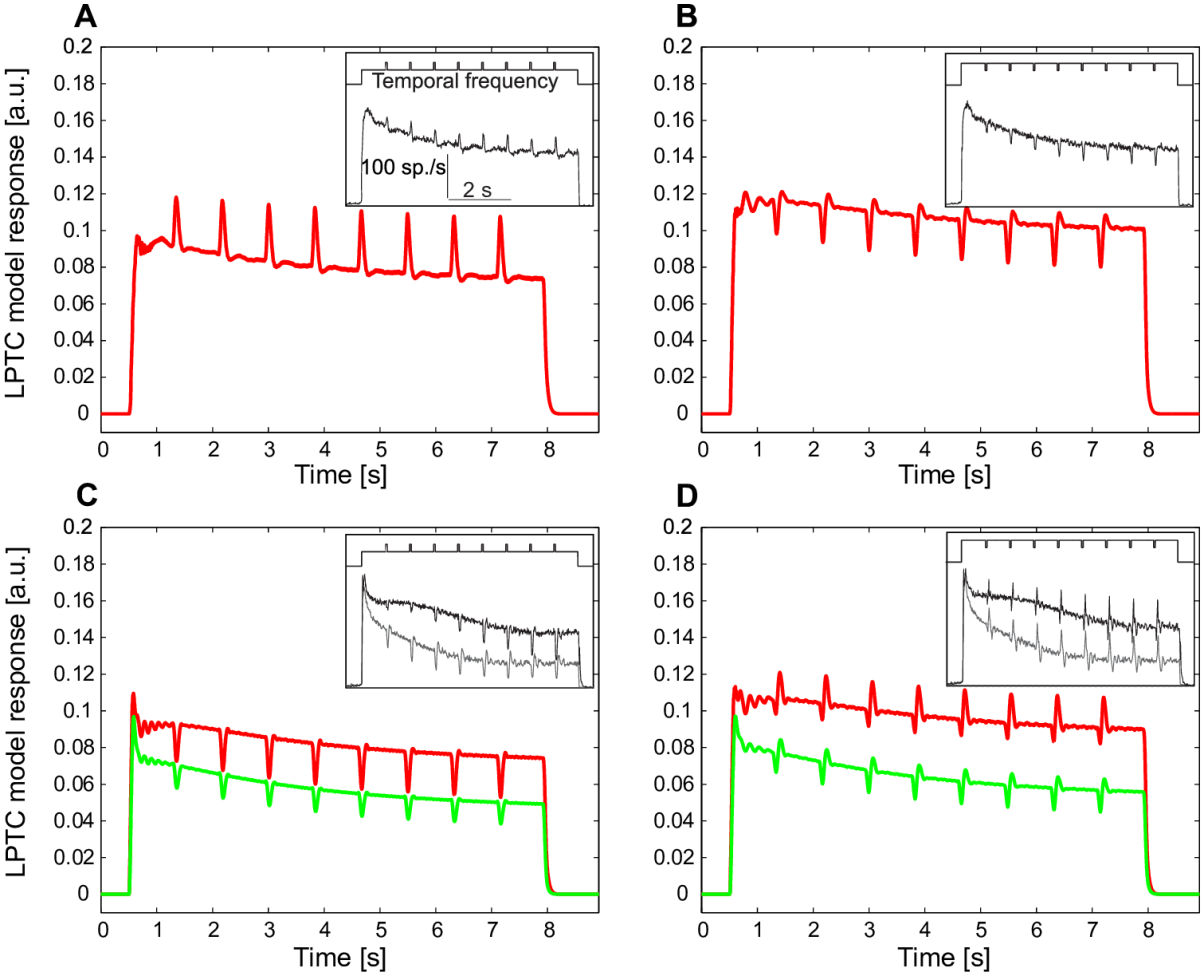
Responses of a model LPTC to constant-velocity motion superimposed by brief velocity transients. (A) LPTC model response (red) to constant motion of a sine-wave grating interspersed with incremental temporal frequency transients (inset, above the LPTC response), in comparison to electrophysiologically determined LPTC response (inset, from Figure 1 of [27]) to the same type of stimulus. (B) Same as (A), however, the velocity transients were decrements. In contrast to (A, B), in which the constant velocity is at the rising slope of the bell-shaped steady-state velocity tuning curve of motion detectors, in (C) the constant background velocity is at the falling slope of the velocity tuning curve, and (D) in the peak region of the bell-shaped tuning curve of motion detectors (red: high brightness contrast of grating, green: low contrast).

**Fig 3:**
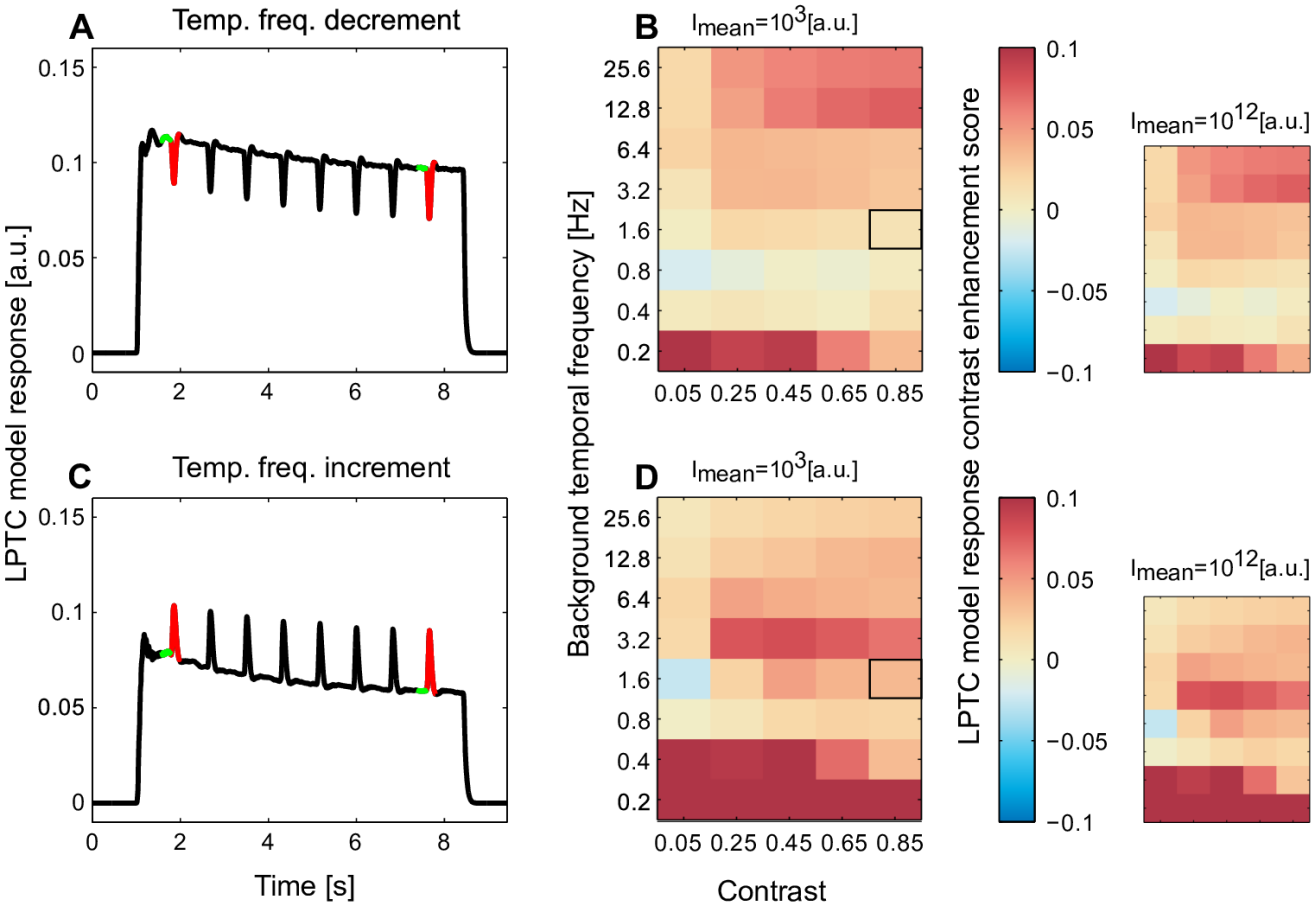
Enhancement of response contrast by motion adaptation over a wide range of stimulus parameters. (A) An example (corresponding to the condition marked by black frame in B) of model response to the same stimulus scheme as in Fig 2 (black), in which the peak response to the temporal frequency transient (red) and the response to the constant background temporal frequency (green) of the first and the last temporal frequency decrements were used to assess whether the response contrast to temporal frequency transients is enhanced by adaptation. (B) The changes of response contrast to temporal frequency transients (see Eq (5), red: enhancement and blue: reduction of response contrast with adaptation) assessed over a wide range of brightness contrasts of the sine-wave grating and the constant temporal frequencies (smaller plots: the same analysis for light conditions brighter by eight decades). (C, D) Same as (A, B), however, with transient temporal frequency increments rather than decrements superimposed on the background motion.

**Fig 4:**
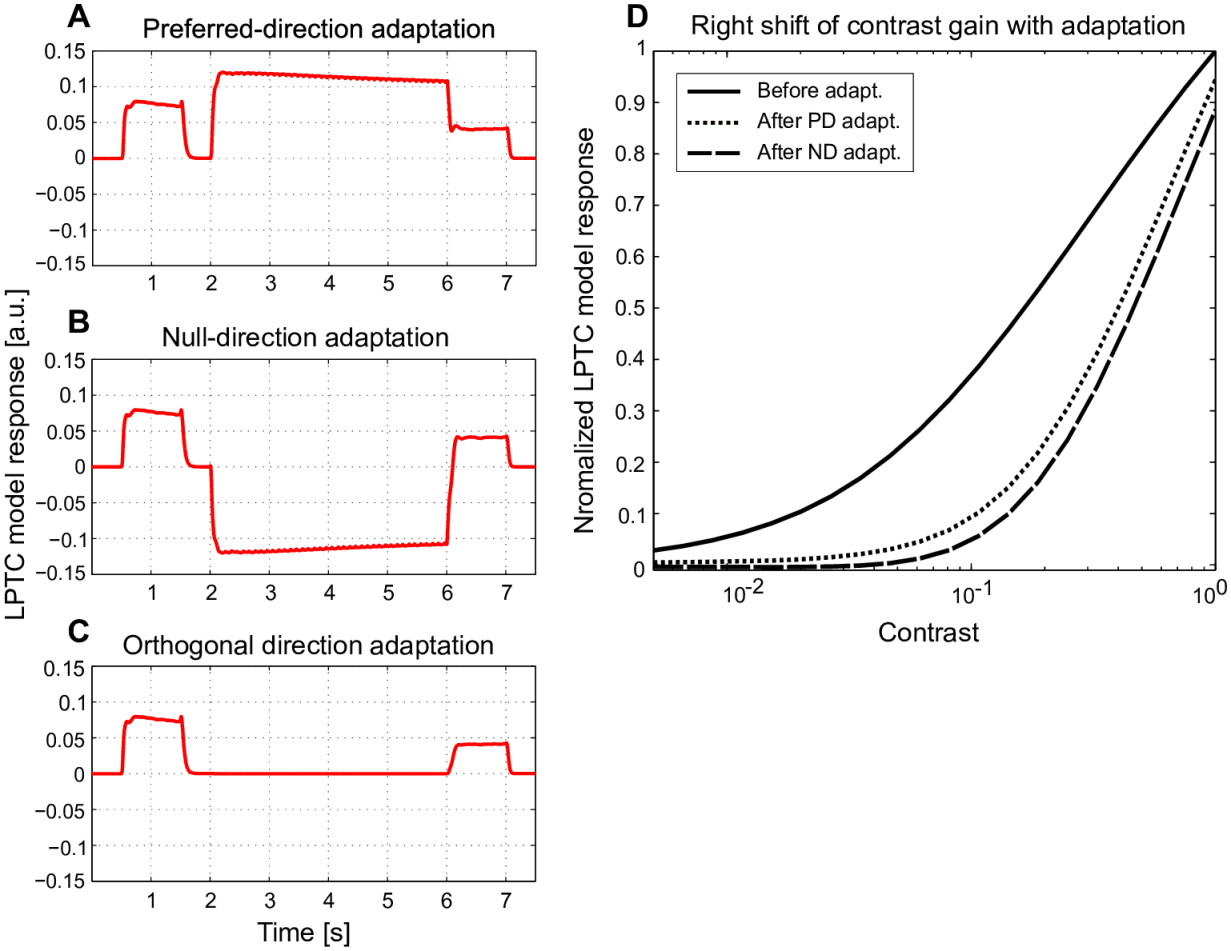
Direction-independent motion adaptation and contrast gain reduction. (A-C) Model responses (red) to 1 s of sine-wave grating motion before and after 4 s of motion adaptation (corresponding LPTC responses to the same type of stimulus, see Figure 2 and 5 in [28]). During the motion adaptation period the sine-wave grating with high contrast and velocity moved in (A) the preferred direction (PD), (B) the null-direction (ND), or (C) an orthogonal direction. (D) For the same stimulus scheme, the brightness contrast of the grating during the reference and test period was systematically varied, and contrast gain was assessed by calculating the normalized response for the first 300 ms of the reference and test period (solid line: contrast gain before motion adaptation, dotted and dashed lines: contrast gain after PD and ND adaptation, see Figure 2 in [28] for corresponding experimental data).

Following the columnar and layered structure of the visual system of flies, our model of the visual motion pathway is composed of successive layers of retinotopic arrays of model photoreceptors (PRs), large monopolar cells (LMCs), EMDs, and of an LPTC integrating the output of large arrays of EMDs (Fig 1). The model parameters were tuned to qualitatively capture adaptive features revealed in previous electrophysiological studies (Fig 2; [26, 27]). The model parameters were determined by systematic search in the chosen parameter range and by selecting the parameter combinations that correspond best to the electrophysiological benchmark data. The model was not only validated for the benchmark data, but also for a wider range of stimulus parameters (Fig 3) and also by using other types of stimuli that were not used for its optimization (Fig 4).

**Fig 5:**
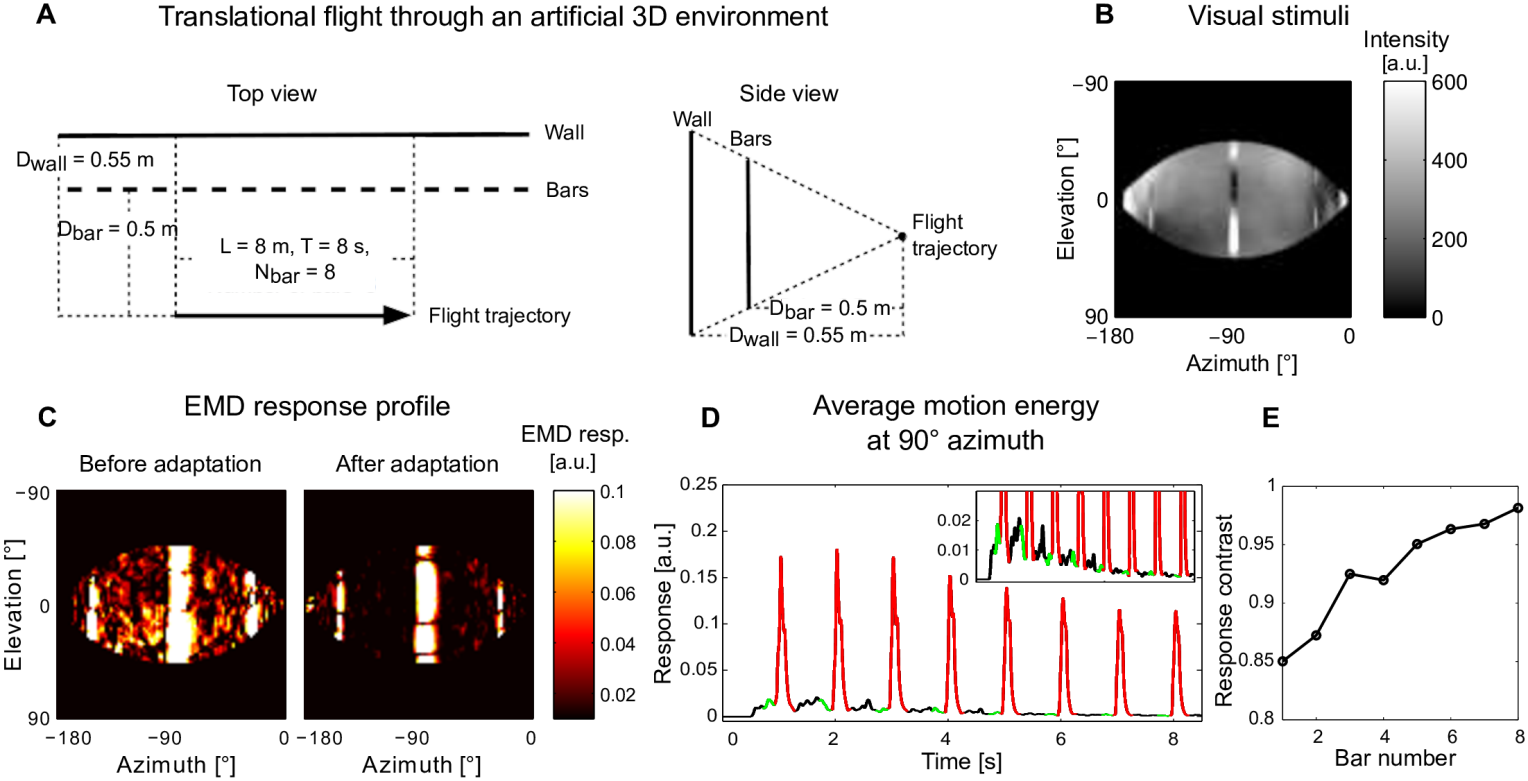
Impact of motion adaptation on spatial vision during translation in an artificial 3D environment. (A) Schematic illustration of spatial layout of the artificial 3D environment and the flight trajectory of an artificial agent translating parallel to a row of bars and a wall behind the bars. (B) The projection of the environment on the left hemisphere of a spherical eye. (C) The EMD response profile before adaptation (as the first bar passing by, left sub-Figure) and after adaptation (as the eighth bar passing by, right sub-Figure). (D) Motion energy averaged across elevation at 90° azimuth as a function of time; the response to the background wall is shown in the inset on a finer scale (red and green: section of response used to assess peak responses to bars and background for the purpose of assessing response contrast). (E) Response contrast between bar and background responses during the passage of each of the eight bars.

**Fig 6:**
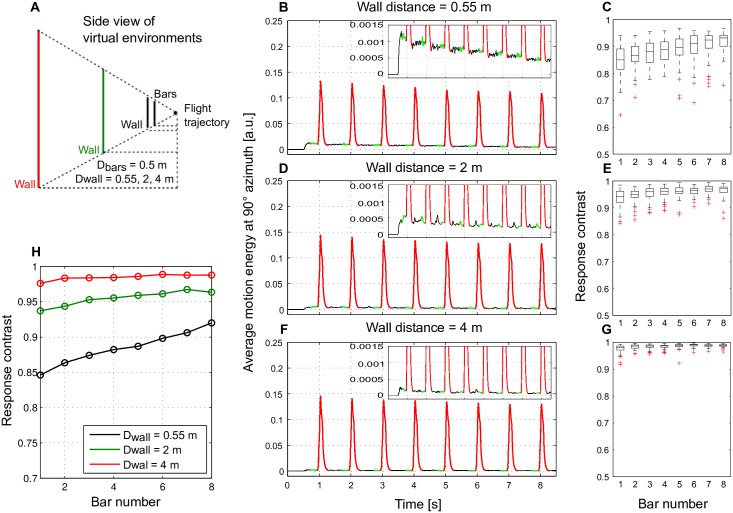
Enhancement of motion detector response contrast with adaptation for different fore- and background depth differences. (A) Schematic of the spatial layout of a 3D environment and the flight trajectory of an artificial agent, the same environmental design as in Fig 5A, however, for three different wall distances in different scenarios (black: wall distance 0.55 m, green: 2 m, red: 4 m). (B) The average motion energy across elevations at 90° azimuth over time as assessed in Fig 5D, however, averaged over 50 different wall and bar patterns. (C) Response contrast between each bar and background response with adaptation. Results obtained from 50 different random wall and bar patterns summarized in box plots (mid-line: median; box: 25–75 percentile: red cross: outlier). (D-G) Same as (B, C), however, with wall distances of 2 m and 4 m, respectively. (H) Averaged response contrast between bar and background as a function of time for all three scenarios with different wall distances over 50 different random wall and bar patterns.

**Fig 7:**
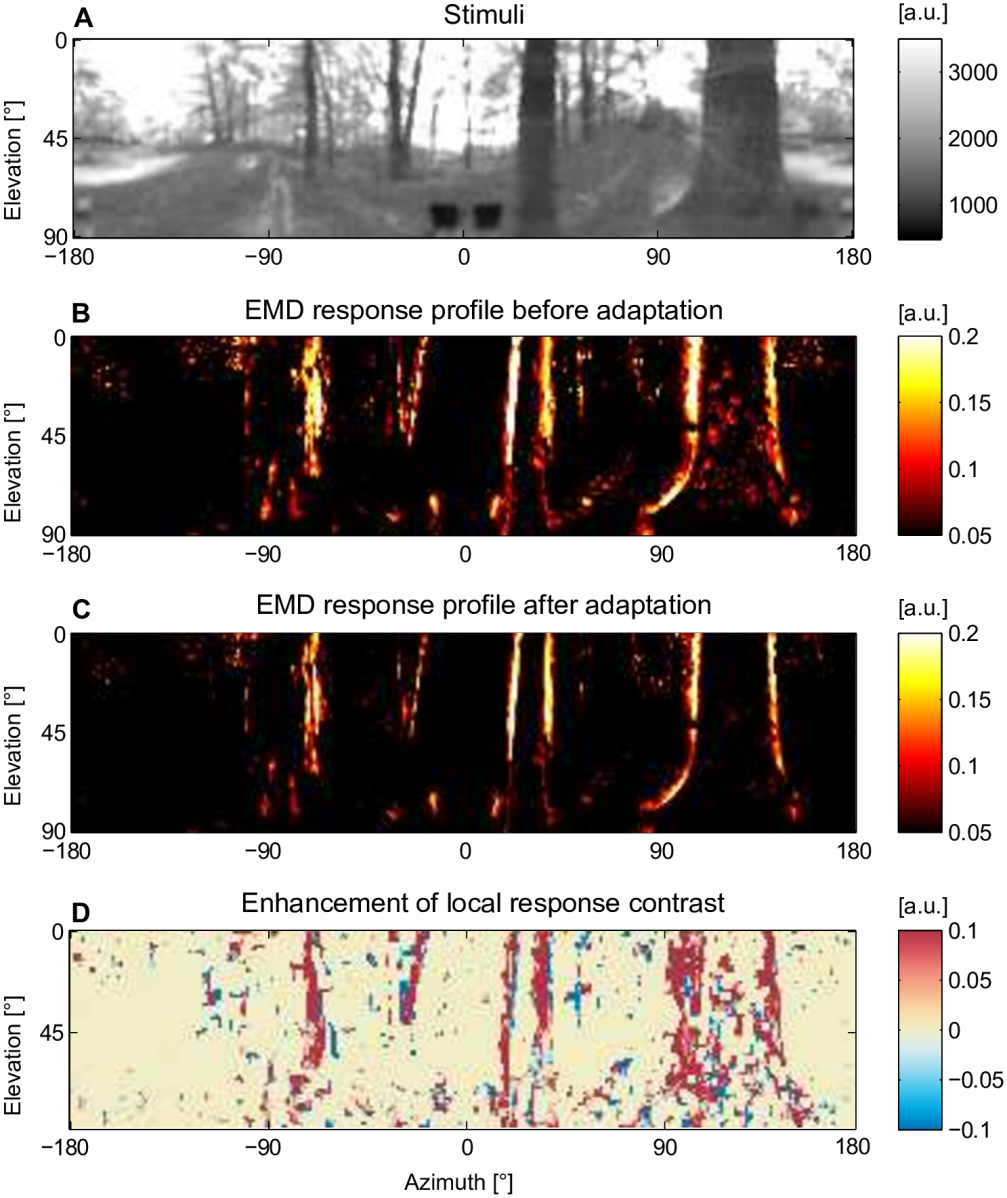
Impact of motion adaptation on the representation of spatial information by arrays of motion detectors during translational flight in a natural cluttered environment. (A) Middle frame from an image sequence mimicking the retinal input during a translational motion in a forest. The whole stimulus sequence is composed of eight repetitions of a 900-ms-translational optic flow sequence. (B) EMD response profile before motion adaptation (in the middle of the first repetition, t = 450 ms) and (C) after motion adaptation (in the middle of the eighth repetition, t = 6750 ms). (D) Assessment of local response contrast changes with adaptation by subtracting the local response contrast of the EMD profile after adaptation (C) from that before adaptation (B) (red: enhancement and blue: attenuation of local EMD response contrast).

**Fig 8:**
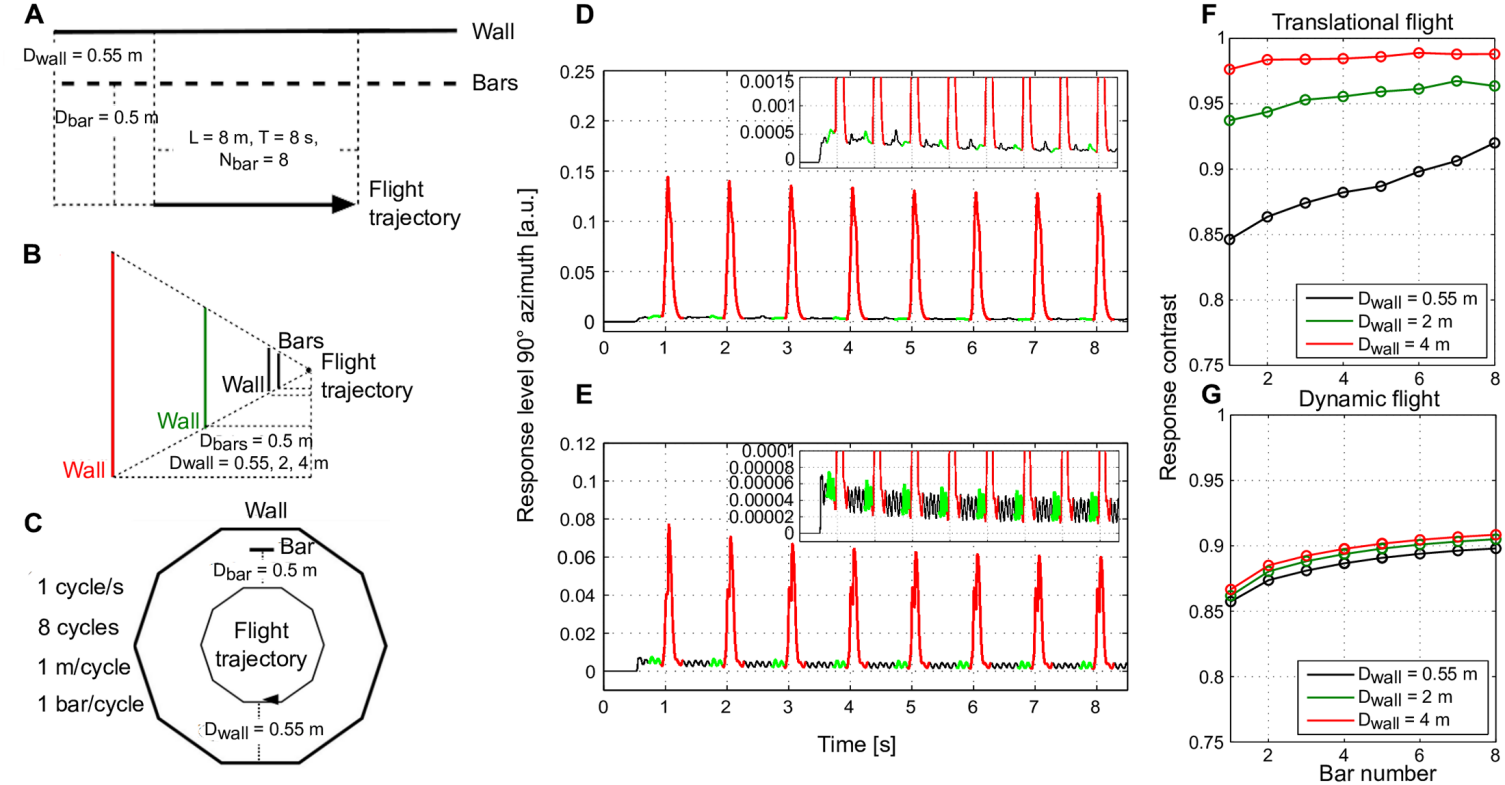
Impact of motion adaptation on spatial vision for semi-natural flight dynamics. Schematic of the spatial layout of a 3D environment and the flight trajectory of an artificial agent with (A) only translational movement or (C) with semi-natural flight consisting of eight cycles of a decagonal trajectory. (B) The same side view as the agent passes by a bar shared between conditions (A) and (C) for all three wall distances tested (black: 0.55 m, green: 2 m, and red: 4 m distance between wall and trajectory). (D) Average motion energy at 90° azimuth over time for the wall distance of 2 m averaged over 50 different wall and bar patterns (same as Fig 6D). (F) Average response contrast (over 50 different wall and bar patterns) between bar and background responses over time for all three wall distances (same as Fig 6H). (E, G) The same analysis as in (D, F), however, under semi-natural flight conditions in the environment as illustrated in (C).

The overall goal of our model analysis was to find out how motion adaptation affects the representation of optic flow-based spatial information by arrays of EMDs. Therefore, we analyzed the responses to optic flow experienced in both virtual and natural 3D environments during constant-velocity motion (Figs 5–7) and by taking the natural flight dynamics of flies into consideration (Fig 8). The structure of the model as well as the stimuli are described in the following.

### Adaptive model of the visual motion pathway

#### Adaptive model of the peripheral visual system

The peripheral visual system consisting of PRs and LMCs was modeled according to Li et al. [22] (Fig 1B). In each input line, the brightness signal is split into two signal branches. One branch is low-pass filtered with a small time constant of 9 ms, leading to a signal that follows even high-frequency intensity fluctuations; the other branch is low-pass filtered with a large time constant of 250 ms, leading to a signal that indicates the current light condition on a much slower timescale. By adding a constant to the latter branch and dividing the output of the fast branch by this signal, a saturation-like transformation function is obtained that shifts adaptively over time according to the current light condition.

$$PR_{resp}=\frac{LP1_{PR}\left(I\right)}{LP2_{PR}\left(I\right)+C_{PR}}$$ (1)

In Eq (1), *I* is the input intensity, *PR*_*resp*_ is the photoreceptor response, *LP*1_*PR*_ and *LP*2_*PR*_ are first-order low-pass filters with small and large time constants, and *C*_*PR*_ is a constant. This adaptive photoreceptor model allows the visual system to operate over eight to ten decades of light intensities [22].

The photoreceptor output is then fed into the LMC model. The LMC model is a first-order high-pass filter that eliminates the information about the average brightness level. This high-pass filtering has been shown to be essential for extracting depth information by the motion detectors at the next processing stage [22]. As an elaboration of our earlier model [22], the LMC output is half-wave rectified and split into an ON and an OFF pathway according to its biological counterparts [33–35]. Furthermore, a saturation-like non-linearity was introduced to the LMC output by dividing the LMC output by the sum of the LMC output and a constant (Fig 1B).

#### Adaptive elementary motion detector model

The LMC output of the ON and OFF pathway, respectively, is fed into the adaptive motion detector model (Fig 1B). This motion detector model is composed of a correlation-type motion detector and an adaptive processing of each half-detector output. The correlation-type motion detector is composed of two mirror-symmetric half-detectors sensitive to motion in opposite directions. Each half-detector detects motion by multiplying the delayed signal from one LMC output with the non-delayed signal from the corresponding neighboring LMC output. This leads to four EMD outputs for each column, two for preferred-direction (PD) motion of ON and OFF signals, respectively, and two for null-direction (ND) motion of ON and OFF signals. Each of the EMD outputs is processed by an adaptive mechanism similar to that of brightness adaptation of the photoreceptors (Eq (1), [22]), namely by dividing a fast signal branch following the fluctuations in the motion signal by a slow signal branch representing pattern velocities on a much slower timescale and embedding in a saturation-like Lipetz-transformation. Since motion adaption takes place on a much longer timescale than brightness adaptation in the peripheral visual system, the ‘fast’ and ‘slow’ time constants are much larger, 20 ms and 4 s, respectively, than the ‘fast’ and ‘slow’ time constants characteristic of brightness adaptation (see above). While the fast branches are the different half-detector outputs after being low-pass filtered with a small time constant, the slow branch is the average of all four half-detector outputs being low-pass filtered with a large time constant (Fig 1B). Adaptation of all four branches by the same slow signal was essential for achieving direction-independent motion adaptation. Moreover, to account for the increase of response transients to velocity changes an adaptive exponent is implemented in each component of the adaptation equation.

$$EMD_{adpt}=\frac{LP1_{EMD}{\left(EMD_{nadpt}\right)}^{a}}{LP2_{EMD}{\left(EMD_{ave}\right)}^{a}+C_{EMD}^{a}}$$ (2)

In Eq (2), *EMD*_*adpt*_ is the adapted EMD response of each branch, *EMD*_*nadpt*_ is the unadapted EMD response corresponding to the response after the multiplication, and *EMD*_*ave*_ is the average *EMD*_*nadpt*_ of all four branches. *LP*1_*EMD*_ and *LP*2_*EMD*_ are low-pass filters, *C*_*EMD*_ is a constant, and *a* is an adaptive exponent adjusted according to *EMD*_*ave*_:

$$\begin{array}{c} \frac{da}{dt}=-\left(a-a_{min}\right)*p_{1}+\left(a_{max}-a\right)*p_{2}*LP2_{EMD}\left(EMD_{ave}\right) \end{array}$$ (3)

In Eq (3), *a*_*max*_ and *a*_*min*_ are the upper and lower boundaries of exponent *a*, *p*_1_ and *p*_2_ are constants determining the speed of recovery and the strength of adaptive modification. *LP*2_*EMD*_(*EMD*_*ave*_) is the unadapted EMD response of all four branches after they were averaged and low-pass filtered. Note that, the temporal frequency tuning of this correlation-type motion detector as well as its adaptive version are bell-shaped (Supplementary S2 Fig), i.e. with increasing stimulus temporal frequency the EMD response first increases, while with a further increase in temporal frequency the EMD response reaches an optimum and then decreases again.

#### LPTC model

For simplicity, we assume that the outputs of the local motion detectors are linearly summated at the next stage of signal processing corresponding to the level of LPTCs (Fig 1B). Here, both half-detectors, i.e. ON and OFF, responding best to preferred-direction motion contribute to the sum with a positive sign, whereas both half-detectors responding best to null-direction motion contribute with a negative sign. The simplification of linearly summating the motion detector outputs instead of implementing a dynamic gain control at this processing stage [36, 37] is justified in the context of the current paper, since the pattern size in all model simulations was kept constant. (Note that, the LPTC model is only used for the model development and characterization (Figs 2–4), while the impact of motion adaptation on spatial vision is being analyzed at the level of adaptive EMD arrays (Figs 5–8)).

### Stimuli for model development and characterization

#### Stimulus set 1

The first stimulus set was characterized by a sine-wave grating moving at a constant velocity (7420 ms) superimposed by eight short (50 ms) velocity transients at regular time intervals (780 ms) (Fig 2 upper panel of insets). Before and after this motion stimulus the grating was stationary for 500 ms, as in the corresponding experiments by Kurtz et al. [27] and Maddess and Laughlin [26]. For convenience, we used in many places the term ‘velocity’ instead of ‘temporal frequency’, i.e. the ratio between the velocity and spatial wavelength. This is justified, because the spatial wavelength was kept constant throughout our simulations and, thus, velocity and temporal frequency are proportional. The transients are either increments in temporal frequency (Fig 2A, from 2 Hz to 4 Hz) or decrements in temporal frequency (Fig 2B, from 4 Hz to 2 Hz); the temporal frequency of the constant background motion was selected to be either smaller (Fig 2A and 2B) or larger (Fig 2C, 8 Hz background to 12 Hz transients) than the optimum of the bell-shaped, steady-state velocity tuning curve of EMDs, or it matched the optimum (Fig 2D, 6 Hz background to 3 Hz transients); the brightness contrast was either high (Fig 2 red, *c* = 0.88) or low (Fig 2 green, *c* = 0.3). For all scenarios (Fig 2A–2D) the sine-wave grating was a 3 × 360 pixel^2^ matrix with average intensity *I*_*mean*_ = 1000 a.u., and spatial wave length λ = 19 pixel. (See [27] for the corresponding parameters used in the electrophysiological experiments.)

In order to assess under which conditions the sensitivity to velocity discontinuities is enhanced by motion adaptation we used the same stimulation scheme as described above and systematically varied the temporal frequency (0.1, 0.2, 0.4, 0.8, 1.6, 3.2, 6.4, 12.8, and 25.6 Hz) and brightness contrast (0.05, 0.25, 0.45, 0.65, and 0.85) of the grating. For each combination of temporal frequency and contrast, the temporal frequency of the transients was either half (Fig 3A and 3B) or twice as large (Fig 3C and 3D) as the background temporal frequency. The model response was calculated for each of these conditions to assess whether the response contrast between the responses to the temporal frequency transients and the responses to constant background motion is enhanced by adaptation. The same stimulus scheme and response analysis was also done at a light level being brighter by eight decades (*I*_*mean*_ = 10^12^ a.u. in Fig 3B and 3D smaller plots in contrast to *I*_*mean*_ = 10^3^ a.u. in Fig 3B and 3D main plots) to test model performance for a wide range of light intensities.

#### Stimulus set 2

Stimulus set 2 was used to test the responses of model LPTCs to a moving grating before and after adaptation with constant-velocity motion of a grating, as used by Harris et al. [28]. Stimulus set 2 was characterized by the following sequence: a homogeneous screen of average brightness (500 ms), a reference stimulus consisting of motion of a sine-wave grating (1 s, *c* = 0.3, 3 Hz), a homogeneous screen of average brightness (50 ms), a long motion adaptation stimulus consisting of a grating of high contrast and constant velocity (4 s, *c* = 0.95, 5 Hz), immediately followed by a test stimulus of the same stimulus parameters as the reference stimulus, followed by a homogeneous screen (500 ms) (Fig 4A–4C). During the adaptation phase the grating moved either in the preferred direction (PD) (Fig 4A), in the null direction (ND) (Fig 4B) or in the orthogonal direction (Fig 4C). The sinewave grating was a 90 × 90 pixel^2^ matrix with average brightness *I*_*mean*_ = 1000 a.u. and spatial wave length of 18 pixels.

Furthermore, under the same stimulus scheme as in Fig 4A and 4B, the contrast of the grating of the reference and test stimulus was varied systematically (20 logarithmically and equally distributed contrast levels between 0.005 and 1) in order to analyze how motion adaptation modifies the contrast gain (Fig 4D, see also Figure 2a in [28]).

### Stimuli for analyzing the role of motion adaptation for representing depth information

#### Visual stimuli generated by translational motion in virtual 3D environments

We used a virtual 3D environment consisting of a wall (1.1 m high, 16 m long, 0.55 m away from the flight trajectory) and a row of bars (5 cm wide, 1 m high, 1 m spacing, 0.5 m away from the flight trajectory) in front of the wall. An agent with one spherical eye (2° spatial resolution) moved parallel to the wall and the row of bars. It passed 1 bar/s during its 8 seconds of translational motion (Fig 5A). The wall and the bars were textured with a random cloud pattern with 1/*f*^2^ statistics (*f* is the spatial frequency). The 3D environments were generated with Open Inventor 1.0 and the visual stimuli experienced by the agent were generated by Cyberfly toolbox developed by Lindemann et al. ([38]; Fig 5B).

The spatial discontinuities between the bars and the background cause discontinuities in retinal velocities during translational motion. In order to compare the impact of motion adaptation on different depth differences we increased the distance between the objects and the wall without changing the distance between the bars and the agent (Fig 6A). If we assume a flight speed of 1 m/s, the distance between the flight trajectory of the agent and the row of bars correspond to 0.5 m, and the walls in different scenarios to 0.55 m, 2 m, and 4 m. We adjusted the size of the wall accordingly to have the same-sized projection of the wall on the retina (Fig 6A). As a result, different spatial scenarios were characterized by the same retinal size of wall texture elements and the bars, but a lower background velocity with increasing wall distance. In order to distinguish the influence of depth transients on the responses from the influence of a specific pattern texture 50 different random cloud walls and bar patterns were included in our analysis.

#### Visual stimuli during translational motion in a natural 3D environment

We also tested a stimulus mimicking what flies experience during translational motion in a cluttered natural environment. By taking a sequence of panoramic photographs along a linear track with the help of a hyperbolic mirror in natural environments (for example in a forest) and applying corresponding rendering methods, image sequences mimicking the retinal image flow during translational motion at 1 m/s in a forest were generated (for details see [21] and published data [39]). The available image sequences recorded in natural environments were too short for investigating the effect of motion adaptation (if we assume a flight speed of 1 m/s, the image sequences correspond to only 900 ms, whereas motion adaptation has a timescale of several seconds [27]). Therefore, we repeated the same image sequence eight times via concatenation. The concatenated image sequences were then fed into our adaptive model of the visual motion pathway. A potential influence of the discontinuity in the scenery due to concatenation was minimized by analyzing the influence of motion adaptation for the frames in the middle of the individual image sequences (Fig 7A).

#### Visual stimuli based on semi-natural flight dynamics

Natural flight of flies consists of segments of translation interspersed with quick saccadic rotations. In order to investigate the impact of motion adaptation on spatial vision under conditions of natural flight dynamics we designed an artificial 3D environment and an artificial flight trajectory taking the dynamics of real flight of flies into account. According to [2] the saccade frequency of free blowfly flights in a cubic box is approximately 10/s; the yaw angle of saccadic turns varies by up to 90°, and the corresponding yaw velocity can reach up to several thousands of degrees per second. Considering these features of real flight dynamics, we generated a semi-realistic flight trajectory by bending each second of the linear translational flight trajectory of a total duration of 8 s, as described, for the pure translational scenario (Fig 8A) into a decagon (Fig 8D). This trajectory was centered in a decagonal flight arena, and the distance from the flight trajectory to the bar and the wall as well as the size of the bar and the height of the wall were kept the same as for the straight trajectory. The bar and the walls were also textured with a random cloud pattern, and the frequency at which a bar was passed by (1 bar/s) was also kept the same. One cycle of the decagonal trajectory was composed of a sequence of 80-ms-pure-translations along the walls of the decagon and 20-ms-pure-rotations that led to a 36° saccadic turn in the corners of the decagon. During the saccadic turns in the corners roll and pitch were kept constant, and the dynamics of yaw-velocity was based on recorded free flight data [3, 38] following the equation:

$$\begin{array}{cc} v_{yaw} & =\frac{36\pi }{180}\frac{G(t)}{\sum_{t=1}^{20}G(t)},\;where\\ & G(t)=\frac{1}{\sigma \sqrt{2\pi }}\text{exp}\left(-\frac{1}{2}{\left(\frac{t-t_{c}}{\sigma }\right)}^{2}\right),t_{c}=10.5,\sigma =3.5,1\leq t\leq 20ms \end{array}$$ (4)

In this way, the saccade frequency, the yaw angle and velocity during saccades were within a realistic range and the total length and duration of the trajectory were identical to the pure translational trajectory.

## Results

### Characterization of motion adaptation by modeling the responses to benchmark stimuli

The adaptive model of the fly visual motion pathway was first tested with visual stimuli that were used in previous electrophysiological studies on fly LPTCs [26–28]. The characteristic responses of the LPTCs were used as a benchmark to adjust the model parameters of our adaptive model.

When presenting a sine-wave grating moving at a constant velocity superimposed by short-velocity increments as in Kurtz et al. [27], both model and LPTC responses decayed over time (Fig 2A). However, the short response increments induced by the increments in velocity were not reduced, but even slightly increased over time (Fig 2A). Both model and cell responses revealed similar adaptive features when the constant-velocity motion was superimposed by velocity decrements: While the overall response amplitude considerably decreased, the response decrements evoked by the velocity decrements were even enhanced over time (Fig 2B). A characteristic feature of both biological and model motion detectors is the bell-shaped steady-state velocity tuning: i.e. the motion detector response increases with velocity up to a certain velocity and then decreases again if the velocity further increases. Similar adaptive features as just described for the rising phase of the velocity-response characteristic (Fig 2A and 2B) were observed when the constant background velocity was on the downward-sloping side of the bell-shaped velocity-response characteristic (Fig 2C) as well at its optimum (Fig 2D). Note that, in Fig 2C transient velocity increments evoked transient response decrements, while in Fig 2D transient velocity decrements evoked fluctuations around background response level. In conclusion, while motion adaptation leads to a reduction of motion-induced responses on a slow timescale, it enhances the relative sensitivity of both LPTCs and the adaptive model of the visual motion pathway to velocity transients under a wide range of stimulus conditions. Since peripheral brightness adaptation implemented in our model, which is in a steady state already within several hundreds of milliseconds [22], it does not much contribute to the described adaptive decay of the background activity and the enhancement of transient response on a timescale of several seconds as is characteristic of motion adaptation.

In order to systematically assess under which conditions the relative sensitivity to velocity increments (Fig 3A and 3B) and decrements (Fig 3C and 3D) is enhanced by adaptation we used the same stimulus scheme as in Fig 2 and systematically varied the velocity and the brightness contrast of the grating (Fig 3B and 3D). The sensitivity to each velocity transient was quantified by calculating the response contrast between the response to the velocity transient and the response to the constant background velocity:

$$C_{resp}=\frac{|R_{bg}-R_{pk}|}{R_{bg}+R_{pk}}$$ (5)

In Eq (5) *C*_*resp*_ is the response contrast; *R*_*bg*_ represents the LPTC model response to the constant background velocity calculated as the average response over 200 ms before the transient response (Fig 3A and 3C, color-coded in green); and *R*_*pk*_ is the peak response within the transient response range (Fig 3A and 3C, color-coded in red). To assess whether the response contrast was enhanced with adaptation we subtracted the response contrast to the first transient from the last one and used this value as an enhancement score (Fig 3B and 3D, color code in black square frames). A positive enhancement score (Fig 3B and 3D, warm colors) indicates an enhancement of response contrast to velocity transients, whereas a negative score (Fig 3B and 3D, cold colors) indicates an attenuation of the response contrast. An enhancement of response contrast to velocity decrements (Fig 3B) as well as increments (Fig 3D) is evident under most examined stimulus conditions of brightness contrast and velocity as revealed by the dominantly warm-colored heat maps. As a consequence of brightness adaptation in the peripheral visual system, this performance was maintained even if the overall pattern brightness was increased by up to 8 decades (Fig 3B and 3D smaller plots).

We tested the model with another type of stimulus as used in a previous electrophysiological study on motion adaptation. As Harris et al. [28] tested the adaptive performance of fly LPTCs, we tested how the response to a velocity step of our adaptive model was affected by adaptation stimuli moving in the preferred direction (PD), the null direction (ND), as well as orthogonal to these directions (Fig 4A–4C). The LPTC model response resembled that of LPTCs in the following qualitative features: The responses after adaptation were considerably smaller than the reference responses before adaptation irrespective of the direction of motion during adaptation (Fig 4A, 4B and 4C). Even if orthogonal pattern motion was used for adaptation, the adaptive effect was present, although both model and LPTCs almost did not respond to the adaptation stimulus (Fig 4C). In the electrophysiological recordings the initial part of the test phase after PD adaptation was less depolarized for a short time interval than that after ND adaptation (see Figure 2a in [28]). This was not the case in the corresponding model response (Fig 4A and 4B). The observed difference between model and experimental data is mainly due to the after-hyperpolarization, which occurs at the LPTC level after a strong depolarization of the cell. Since our present study focuses on the impact of motion adaptation on the EMD-level, the after-hyperpolarization generated in the postsynaptic LPTC has not been taken into account.

Harris et al. [28] further assessed the modulation of contrast gain by motion adaptation (see Figure 2a in [28]) by systematically varying the brightness contrast of the reference and test stimulus and comparing response amplitudes before and after motion adaptation. Part of the response characteristics revealed in their study can be explained by our model, such as the rightward-shift of the contrast-gain curve after motion adaptation (Fig 4D). Our model successfully accounts for the reduction of the contrast gain after both PD adaptation and ND adaptation. However, our model does not explain two other response characteristics described by Harris et al. [28], namely the after-hyperpolarization following PD motion adaptation and the corresponding reduction of the output range of the cell (Fig 4D). Both response characteristics have been concluded to occur at the LPTC level [40] which is not covered by our current model.

### Potential significance of motion adaptation for spatial vision

The above model was used to investigate the impact of motion adaptation on the representation of spatial information at the level of arrays of motion detectors. This was done by simulating the visual input as experienced during translational motion in both virtual 3D environments (Figs 5 and 6) and cluttered natural 3D environments (Fig 7), employing pure translational motion (Figs 5, 6 and 7) or mimicking natural flight dynamics of flies (Fig 8).

#### Translational motion in virtual and natural 3D environments

According to available electrophysiological data and our model simulations, motion adaptation can enhance the relative sensitivity to discontinuities in the motion stimulus, while reducing the overall response to sustained motion (Fig 2). This feature can potentially favor optic flow-based spatial vision, since during translational motion depth contours generate discontinuities in the optic flow profile, which might be enhanced as a consequence of motion adaptation. In order to test this hypothesis we first moved a virtual agent parallel to a row of bars in front of a wall (Fig 5A). The environment projected on the left eye is illustrated in Fig 5B.

We used the resulting motion sequence as the input to our model of the visual motion pathway and compared the response profile of the retinotopic EMD arrays before (Fig 5C left) and after (Fig 5C right) motion adaptation, i.e. when the first bar vs. when the last bar was passing the lateral part of the visual field. The responses to both the bars and the background wall were generally reduced after adaptation. However, as a consequence of motion adaptation, the response to the background wall pattern was much more reduced in comparison with the response to the bars making the bars more salient in the overall response profile of the EMDs (Fig 5C).

In order to quantify this impression we assessed the sensitivity to the depth discontinuities in the following way: First, we combined the temporal development of EMD responses to bars and background wall to one variable. To this end, we chose the lateral (azimuth = 90°) part of the visual field for our response analysis, because for geometric reasons bars passing the visual field at 90° azimuth led to the strongest responses and covered most of the vertical extent of the visual field. We then calculated the average of the motion energy (i.e. absolute value of EMD responses) at 90° azimuth over time (Fig 5D), which represents both bar and wall responses over time. Finally, based on these time-dependent bar and wall responses we calculated the response contrast according to Eq (5) for each of the eight consecutive bars and the corresponding wall sections (Fig 5E). Due to the strong reduction of background activity (Fig 5D inset), the response contrast increased almost monotonically with adaptation (Fig 5E).

To investigate the impact of the distance (and consequently retinal velocity) differences between the bars and the wall the wall was placed at a distance of 0.55 m, 2 m and 4 m from the flight trajectory in different scenarios, while the bars were kept unchanged at 0.5 m from the flight trajectory (Fig 6A). Moreover, to reduce potential effects of a specific cloud pattern we assessed the bar and wall responses (Fig 6B, 6D and 6F) and the response contrast between the bars and the wall (Fig 6C, 6E and 6G) as in Fig 5D and 5E for 50 random wall and bar patterns and averaged across textures (Fig 6B, 6D, 6F and 6H). The response contrast evoked by the bars increased for all wall distances tested (Fig 6H) as a consequence of a strong reduction of background wall responses with adaptation (Fig 6B, 6D and 6F insets). This effect was the more pronounced the closer the wall was to the bars and, thus, the smaller the response contrast was before motion adaptation (Fig 6H). Thus, motion adaptation enhances the sensitivity of the motion detection system to depth discontinuities.

This conclusion was further corroborated with more realistic stimuli mimicking the visual input during translational motion in a forest (Fig 7A). Because the original image sequence has a duration of only 900 ms (assuming 1 m/s flight speed) which is too short for investigating motion adaptation, the original image sequence was concatenated eight times. In order to see how motion adaptation affects the representation of the environment by arrays of motion detectors the response profiles of EMDs before adaptation (i.e. in the middle of the first repetition of a translational trajectory, Fig 7B) was compared with that after adaptation (i.e. in the middle of the eighth repetition, Fig 7C). Similar, but less prominent effects as in Fig 5C can be observed here: Motion adaptation led to a larger reduction of the responses to background structures than to the contours of nearby tree trunks, which makes the nearby tree trunks more salient. In order to better assess the influence of motion adaptation on signal representation by EMD arrays we calculated the local response contrast of the EMD response profile before and after motion adaptation and subtracted the local response contrast profile after adaptation from that before adaptation (Fig 7D). The red color indicates regions in the environment where the local response contrast was enhanced by adaptation, which corresponds mainly to the contours of nearby tree trunks.

#### During semi-natural flight

According to our simulation results, motion adaptation enhances the segregation of foreground objects from their background if an agent performs pure translational motion (Figs 5–7). However, the translational periods of insect flight are never as long, but frequently interspersed with fast saccadic turns. To test whether the image flow induced by saccadic turns affects our conclusion that the representation of nearby contours is enhanced by motion adaptation, we designed a semi-natural flight trajectory that takes several features of natural flight dynamics into account [2]. Furthermore, we shaped the trajectory and the virtual 3D environments into decagons, so that the visual stimulus was as similar as possible to that used to obtain the results shown in Fig 6 (Fig 8A–8C). The same response analysis was performed as in Fig 6. Under such semi-natural flight conditions (Fig 8E and 8G), the background activity was dominated by the rotational response. This background activity was strong and rather independent of wall distance. Therefore, the response contrast curves increased with motion adaptation, but with a relatively shallow slope. Moreover, the response amplitudes did not differ as much for different wall distances as those obtained during linear motion without saccadic turns interspersed (compare Fig 8F and 8G).

Without saccadic turns, the response contrast between the bars and the background increased most when the distance between wall and bars was smallest and, accordingly, the background and bar responses most similar. Thus, the adaptation is most effective when an enhancement of the response contrast is particularly relevant to segregate objects from their background. However, this characteristic is hardly visible under semi-naturalistic conditions, in which large responses were induced by saccades. However, the general qualitative feature of enhanced response contrast between nearby objects and background with motion adaptation was maintained even with saccades, though to a much smaller extent. There is evidence that responses to saccadic turns are suppressed in visual neurons (measured in LPTCs) by efference copy signals [41, 42]. Although the exact location of the target of the efference copy is not yet clear, this mechanism can potentially counteract the above mentioned detrimental effects of saccades on the consequences of motion adaptation.

## Discussion

The present study shows by model simulations that local motion adaptation as observed in the fly visual pathway facilitates optic flow-based spatial vision by enhancing the representation of nearby objects in the response profile of arrays of local motion detectors. This is due to the fact that motion adaptation strongly reduces the responses to constant or slowly-varying background velocities, while maintaining or even enhancing the responses to velocity discontinuities. Because discontinuities in the optic flow in different regions of the visual field are caused by discontinuities in the depth structure of the environment during translational locomotion, the enhanced sensitivity to optic flow discontinuities is concluded to improve the representation of the depth structure.

The above conclusion has been obtained by model simulations with a novel adaptive model of the visual motion pathway of flies (Fig 1). The model was developed mainly based on response characteristics of fly motion sensitive neurons recorded in previous studies ([26, 27], Fig 2). In this model, motion adaptation is accomplished by a modified version of a mechanism that has previously been used to model brightness adaptation of photoreceptors [22], although adaptation at the different processing stages operates on very different timescales, with motion adaptation being much slower than brightness adaptation. Motion adaptation is based on a divisive interaction of the relatively fast output signal of each half-detector with a much slower branch. The slower branch reflects the direction-independent motion energy by combining the temporally low-pass filtered output of all half-detectors at this retinal location irrespective of their preferred direction. Decisive for motion adaptation to enhance the response to optic flow discontinuities is an adaptation of the exponent of each component of the division by the direction-independent motion energy level (see Eqs (1)–(3)). The adaptive model of the visual motion pathway does not only account for the benchmark response features of fly motion sensitive neurons under a wide range of stimulus conditions (Figs 2 and 3), but also reproduces the direction-independent component of motion adaptation (Fig 4A–4C; [28]) and the contrast gain reduction (Fig 4D; [28]) observed in the fly nervous system.

With this adaptive model of the visual motion pathway, we could show that during translational motion in artificial (Figs 5 and 6) and in cluttered natural (Fig 7) 3D environments motion adaptation may enhance the sensitivity to velocity discontinuities in the retinal image induced by nearby objects. We could further show that this conclusion remains even valid under dynamic conditions mimicking the free flight behavior of insects (Fig 8).

### Adaptive model of the insect visual motion pathway

Several previous modeling studies have been dedicated to explain motion adaptation in the fly visual pathway at the level of LPTCs [43–46] and to decompose the components of the mechanisms involved [28].

Clifford and Ibbotson [45] explained the reduction of the cell response to constant-velocity motion, while maintaining or enhancing sensitivity to brief velocity changes [26, 27] by adaptive changes of the EMD low-pass filter time constant by feedback control. This time constant is specific for the motion detection circuit and, especially, for determining its velocity tuning. In contrast, the adaptation mechanism proposed in the present study is a more general-purpose feed-forward adaptive model. The computational principle underlying this mechanism can be used at different stages of the visual pathway to explain, after adjustment of the time constants to the particular functional needs, brightness adaptation of photoreceptors as well as motion adaptation of the motion detection circuits. This simple adaptation mechanism does not only explain the enhancement of response contrast with motion adaptation for a wide range of test conditions (Figs 2 and 3). It also explains that motion adaptation in fly LPTCs is to a large extent direction-independent (Fig 4; [28, 44]), and reproduces the reduction of contrast gain (Fig 4; [28]). We could not validate these features (Figs 2, 3 and 4) by re-implementing and testing the model of Clifford [45] (see [47]).

Harris et al. [28] analyzed the adaptive properties of LPTCs by confronting them with grating motion before and after a period of motion adaptation in PD, ND or in the orthogonal direction. They attributed motion adaptation observed in LPTCs to three components: (1) a motion-dependent, but direction-independent contrast gain reduction, (2) a strong direction-selective after-hyperpolarization, and (3) an activity-dependent reduction of the response range. Amongst these adaptive components, our model can account for the direction-independent contrast gain reduction (Fig 4). The other two components of motion adaptation characterized by Harris et al. [28] are not covered by the present model. This finding is in line with the conclusion that these components of motion adaptation have their origin post-synaptic to the EMDs at the LPTC level [40]. As pointed out above, it has not been the goal of the present study to model LPTCs, but to study the impact of local motion adaptation on the signal representation of environmental information at the level of EMD arrays. However, in principle, depending on the signal used to adapt each branch of the half- detector, this model can be adjusted to also account for direction-dependent motion adaptation.

Another modeling study on motion adaptation of LPTCs attempted to explain a different response feature of LPTCs, i.e. the shortening of the response transients induced by motion steps and motion impulses after adaptation [29, 46]. This feature might potentially further enhance the representation of discontinuities in the optic flow pattern by increasing the temporal resolution of motion detectors. On the other hand, this model based on adapting time constants of filters in the cross-branches of the EMDs before the multiplication stage [46] cannot explain the adaptive benchmark features examined in this study (own results based on a reimplementation of the model of [46]; Supplementary S1 Fig).

Since motion adaptation in our model was realized at the output of the EMD half-detectors rather than by interfering with motion computation itself, this adaptive mechanism could also be applied at the output of other types of motion detector models such as recently published motion detector models combining preferred-direction enhancement and anti-preferred direction inhibition [10].

### Functional significance of local motion adaptation at EMDs

It was already in the fifties of the last century that each stage of signal processing in nervous systems had been suggested to reduce redundancy in order to efficiently use the limited information capacity of neurons and to extract eventually ecologically relevant information [48]. Given the limited coding capacity of all processing stages of a nervous system, it is expected for each layer of neurons to be adaptive, i.e. to be able to adjust its input-output relationship according to recent input history. Examples from insect visual systems (but restricted neither to the visual modality nor to insects [49–51]) are brightness adaptation in photoreceptors and LMCs [52, 53], motion adaptation at the level of local motion detectors (although measured in large-field motion sensitive cells, [26, 27, 29, 43, 54]), and wide-field motion adaptation at the level of LPTCs [28, 40]. It is generally assumed from the perspective of information theory that adaptive coding provides the advantage of an efficient use of the coding capacity of neural circuits by removing redundant (i.e. unchanging or only slowly changing) signals based on the recent input history [49, 50]. Redundancy reduction can increase information transmission [23, 24] and save encoding energy [25, 55].

There have been several studies revealing adaptive features based on electrophysiological experiments on LPTCs using various system-analytical stimuli [26–29, 43], and a major component of the adaptive mechanisms is suggested to occur locally pre-synaptic to the LPTCs [26, 29, 43]. However, how local motion adaptation affects signal representation in the responses of motion detector arrays during flight in the three dimensional world has by now only been analyzed experimentally in an indirect way at the level of LPTCs [30, 31], but due to methodological constraints not at the level of the array of their pre-synaptic local input elements. With our adaptive model of the visual motion pathway, it was possible to analyze the impact of local motion adaptation on the signal representation at EMD arrays, at least by simulation approaches. In this way, we found that, as a consequence of motion adaptation, the representation of foreground objects in an environment is much more salient at the EMD output than the EMD responses to the background clutter (Figs 5–7). Consistent with the experimental results on LPTCs [31], we could show that this segregation of foreground objects from background clutter is maintained, even if translational flights were interspersed with fast saccades, as are the characteristic of insect flight (Fig 8). However, saccades interspersed between translational self-motion segments of the agent attenuate the enhancement of the response contrast between fore- and background and its distance-dependency. This detrimental influence of saccades on representing spatial information by movement detectors may be counteracted by the experimentally established efference copy signals that were found to suppress saccade-driven visual motion responses [41, 42].

What is the functional significance of an enhancement of nearby contours at the movement detector output resulting from motion adaptation? This question cannot yet be answered, because not much is known about how the output of EMD arrays, apart from being LPTC input, is processed. Furthermore, closed-loop control as is characteristic of most behaviors may add complexity to our understanding of the role of local motion adaptation. If the enhancement measured in our model simulations is sufficient to substantially change the detectability of objects is hard to assess without making assumptions on the signal-to-noise situation in a real system and the structure of the following processing steps.

In principle, the information provided by motion detector arrays during self-motion may serve later-stage signal processing subserving a wide range of behavioral tasks, such as (1) optic flow-based spatial vision which is important for detecting objects [56], collision avoidance [57, 58] and landing [59, 60], (2) gaze stabilization during locomotion [2, 3, 61], (3) flight speed control [57, 62] and (4) visual odometry [63, 64]. The impact of motion adaptation on signal processing in these behavioral contexts is still not clear. However, one potentially important aspect is that local motion adaptation at the EMD level is largely direction-independent ([28]; Fig 4). This feature could be functionally important in maintaining equal adaptive states and, thus, equal sensitivity of local motion detectors with different preferred directions. If the sensitivity of differently aligned motion detectors is changed by an adaptive mechanism depending on the direction of motion, the population responses of such detectors would indicate different directions of local motion in response to a given motion direction-depending stimulus history. Thus, direction-independent adaptation might be important in behavioral contexts where a correct representation of local motion direction is essential.

Although this model study is based on the electrophysiological data and flight data from blowflies, there is no reason why the adaptive model and the conclusions about how local motion adaptation enhances the segregation of foreground objects from their cluttered background in optic flow-based spatial vision should be restricted to flies. Moreover, the model may also be useful for implementing artificial motion vision systems.

## Supporting information

S1 Fig Characterization of Borst-Reisenmann-Haag model. LPTC model response (based on reimplementation of model suggested in [46]) to (A) transient sine-wave grating before and after motion adaptation with sine-wave grating motion in preferred direction and (B) constant motion of sine-wave grating interspersed with eight transient velocity increments (as in Fig 2A). See Figure 4 in [29] and Figure 1 in [27] for corresponding electrophysiological data.
(TIF)

S2 Fig Temporal frequency tunning of the LPTC model. Temporal frequency tunning without (A) and with (B) motion adaptation (see Fig 1). There is no substantial shift in the velocity tuning with additional modeling of motion adaptation.
(TIF)
