# Supplementary figures and images for: Local motion adaptation enhances the representation of spatial structure at EMD arrays
Source: PLoS Comput Biol. 2017 Dec 27;13(12):e1005919. doi: 10.1371/journal.pcbi.1005919 (PMC5760083; doi:10.1371/journal.pcbi.1005919)

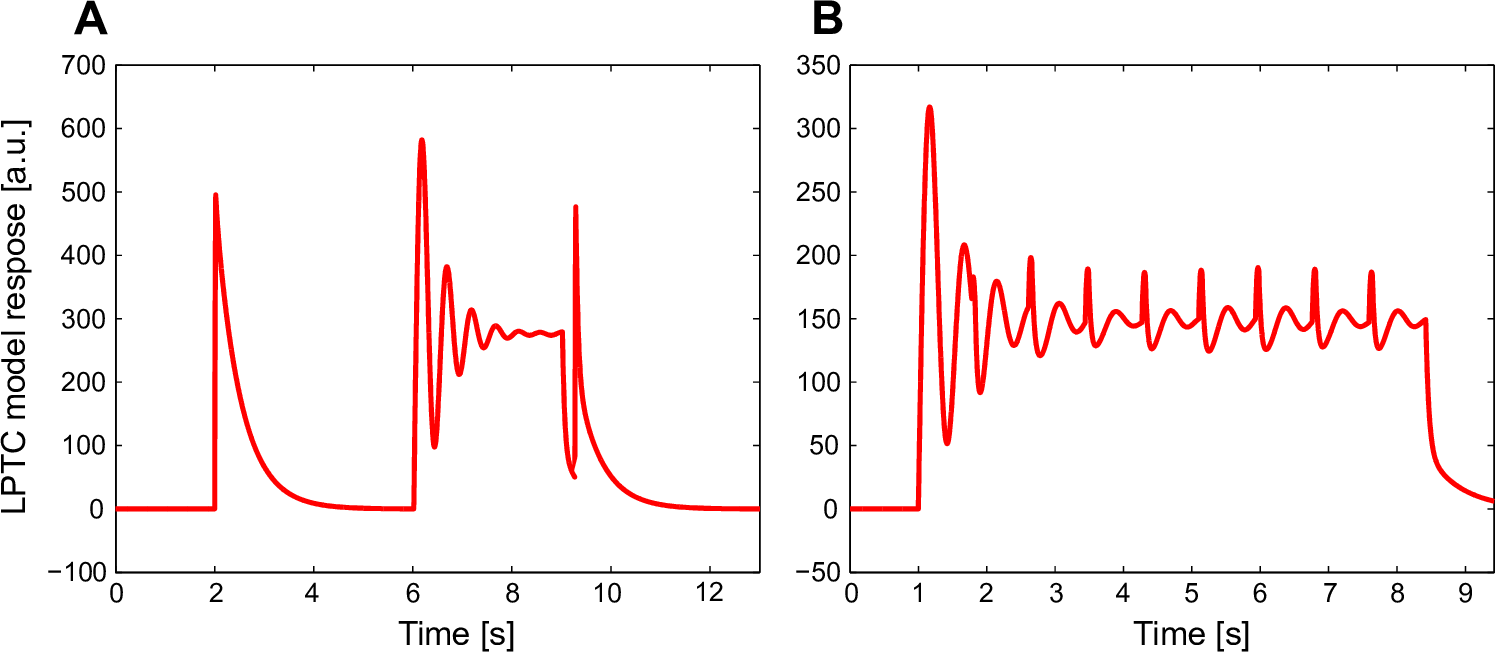

Supplement: S1 Fig — LPTC model response (based on reimplementation of model suggested in [46]) to (A) transient sine-wave grating before and after motion adaptation with sine-wave grating motion in preferred direction and (B) constant motion of sine-wave grating interspersed with eight transient velocity increments (as in Fig 2A). See Figure 4 in [29] and Figure 1 in [27] for corresponding electrophysiological data. (TIF) [file pcbi.1005919.s001.tif]

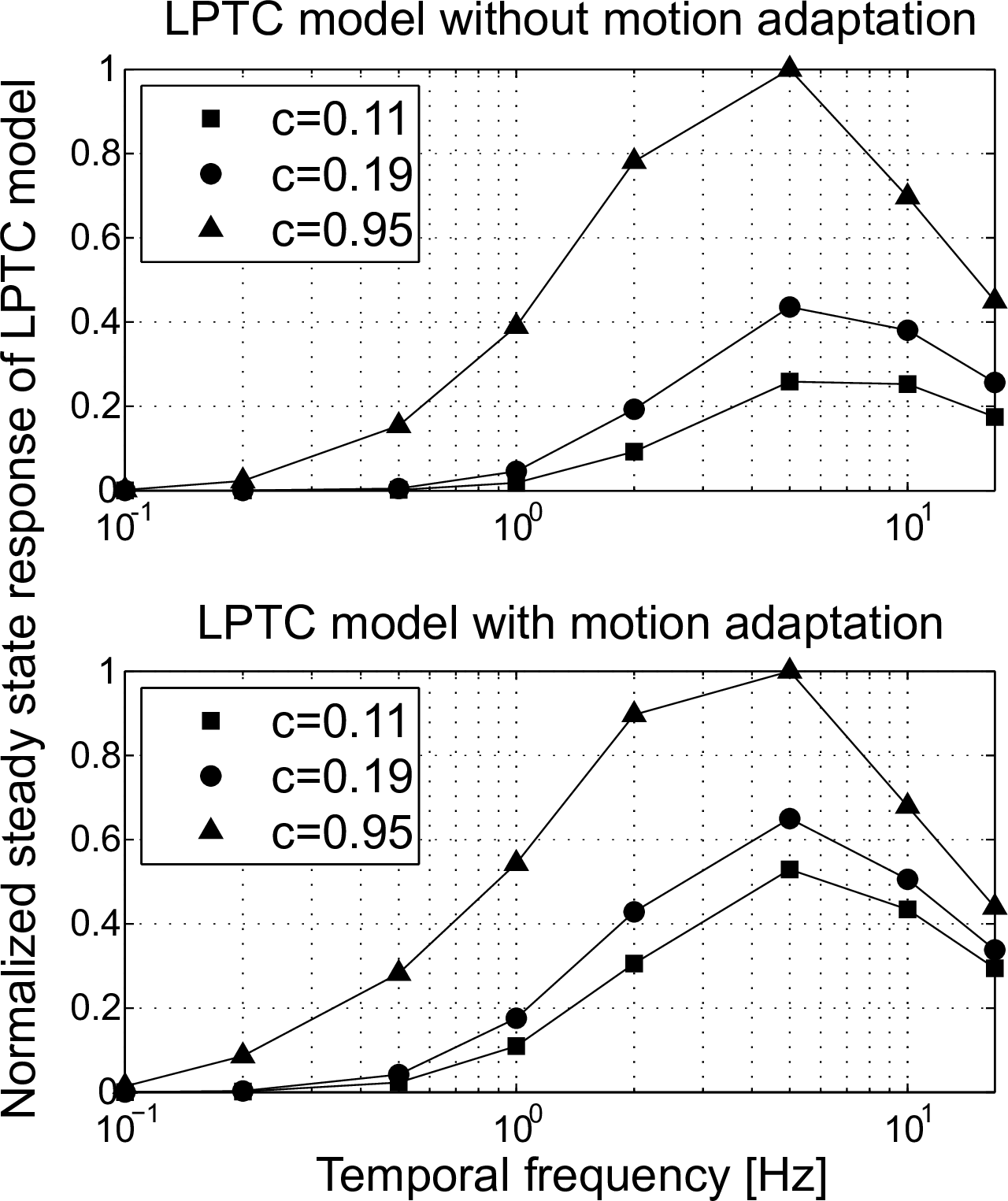

Supplement: S2 Fig — Temporal frequency tunning without (A) and with (B) motion adaptation (see Fig 1). There is no substantial shift in the velocity tuning with additional modeling of motion adaptation. (TIF) [file pcbi.1005919.s002.tif]
